# Effectiveness of dynamic neuromuscular stabilization-based interventions for nonspecific low back pain: a systematic review and meta-analysis

**DOI:** 10.3389/fmed.2026.1926914

**Published:** 2026-09-28

**Authors:** Yuanyuan Ma, Yonghuan Chen, Jae Chul Seo, Hyun-Tae Kim, Jing Ma, Chengzhu Jin

**Affiliations:** 1 Department of Physical Education, Beijing Wuzi University, Beijing, China; 2 Department of Marine Sports, Pukyong National University, Busan, Republic of Korea; 3 College of Humanities and Social Sciences, Pukyong National University, Busan, Republic of Korea; 4 Marxist College, Center of International Development and Cooperation of Northeast Asian Countries, Dalian University of Technology, Dalian, China; 5 Physical Education College, Yanbian University, YanJi, China

**Keywords:** dynamic neuromuscular stabilization, exercise therapy, meta-analysis, nonspecific low back pain, sports rehabilitation

## Abstract

**Introduction:**

Nonspecific low back pain (NLBP) is a leading cause of disability and imposes substantial public health and socioeconomic burdens. Dynamic Neuromuscular Stabilization (DNS) is increasingly used in NLBP rehabilitation, but quantitative evidence of its effects remains limited. We systematically reviewed and meta-analyzed controlled trials evaluating DNS-based interventions for NLBP.

**Methods:**

PubMed, Web of Science, Scopus, the Cochrane Library, and CNKI were searched. Two reviewers independently screened studies and extracted data. Random-effects meta-analysis, subgroup analyses, and leave-one-out analyses were performed using Review Manager 5.4 and Stata 17.0. Potential small-study effects were examined using Begg's rank-correlation test.

**Results:**

Eight studies involving 289 participants were included. The pooled analysis favored DNS-based interventions for pain-related outcomes (SMD = −1.17, 95% CI: −1.85 to −0.48), but heterogeneity was substantial (I^2^ = 85%). Subgroup analyses by intervention strategy, duration, weekly frequency, and session duration did not clearly account for the heterogeneity and were considered exploratory. Begg's test yielded *p* = 0.063, but the assessment of publication bias and small-study effects was inconclusive given the limited evidence base. Leave-one-out analysis indicated that no individual study alone materially altered the pooled estimate, but it did not resolve the remaining clinical and methodological uncertainties.

**Discussion:**

DNS-based interventions may reduce pain-related outcomes in individuals with NLBP; however, the magnitude, clinical importance, and generalizability of the effect remain uncertain. Adequately powered, high-quality RCTs using standardized DNS protocols, clearly defined comparators, and consistent pain-specific outcomes are needed.

**Systematic Review Registration:**

https://www.crd.york.ac.uk/PROSPERO/, PROSPERO (No. CRD420251152728).

## Introduction

The spine, as the central axis of the human body, plays a crucial role in maintaining postural stability, bearing loads, absorbing impact, supporting movement, and protecting internal organs (1). However, with increasing life stress and insufficient physical activity, spinal disorders have shown a rising prevalence and are regarded as a “disease of civilization” (2). NLBP is a syndrome of unknown etiology, primarily characterized by persistent pain located above the gluteal folds, below the costal margin, and between the mid-axillary lines on both sides, with or without radiation to the thighs (3). When symptoms persist for more than 12 weeks, the condition is classified as chronic NLBP (4). NLBP is prevalent across all age groups, with incidence increasing with advancing age (5). The overall prevalence of NLBP is approximately 23%, making it one of the leading global public health concerns and a major cause of disability worldwide (6).

The pathogenesis of NLBP is complex and multifactorial, involving biomechanical factors such as previous injury, excessive physical labor, prolonged static postures, and frequent forward bending, as well as psychosocial influences including psychological and emotional factors (7). Due to the absence of a clear pathoanatomical basis, treatment of NLBP primarily aims to relieve pain and its associated consequences. Common approaches include patient education and reassurance, analgesic medication, non-pharmacological interventions, and reassessment when necessary (8). Current clinical guidelines further emphasize that pharmacological treatment should not be the first-line option. Instead, management should focus on patient self-care, supplemented by physical therapy and psychological interventions, with particular attention to the critical role of exercise therapy in improving NLBP (9).

Recent studies have indicated that patients with NLBP often present with complex impairments in muscle structure and function. Specifically, atrophy of the multifidus and paraspinal muscles has been observed, whereas the erector spinae do not exhibit similar alterations (10). Further evidence suggests delayed activation and dysfunction of the deep multifidus and transversus abdominis muscles in these patients (11). In addition, some studies have reported increased susceptibility of the respiratory muscles to fatigue, which further compromises the stability and performance of the lumbopelvic core musculature (12). A systematic review and meta-analysis also highlighted that reduced lower-limb muscle strength may be a potential contributing factor to NLBP, with significantly lower strength observed in the hip abductors, extensors, and knee extensors compared to healthy controls (13). Therefore, training programs that simultaneously target both muscular and respiratory function may offer greater clinical benefits in reducing NLBP than conventional treatment approaches.

Dynamic Neuromuscular Stabilization (DNS) is a functional rehabilitation method based on developmental kinesiology, with its core principle centered on evaluating and restoring motor system function through movement control patterns governed by the central nervous system (14). This approach emphasizes the critical role of early infant motor patterns in establishing normal motor control and postural stability (15). Researchers have suggested that damage to the neural networks between the brainstem and spinal cord may disrupt central regulation of motor function, thereby inducing biomechanical abnormalities and pathological manifestations such as low back pain (16). DNS-based training is therefore designed to re-regulate the nervous system through a series of specific postures and exercises, with the goal of restoring optimal motor patterns (17). Its primary components include core stability, functional training, balance and coordination, breathing control, and neuromuscular regulation (14). Compared with traditional interventions such as ultrasound therapy, conventional strength training, and stretching exercises, DNS enables rehabilitation practitioners to apply targeted pressure to specific regions, thereby providing afferent stimulation to adjust breathing patterns and activate genetically encoded motor programs of the central nervous system (18).

Findings from a randomized controlled trial demonstrated that patients who underwent a six-week DNS intervention showed significant improvements in movement patterns and balance performance (19), while a recent scoping review suggested that DNS-based training may enhance core muscle activation and alleviate pain-related symptoms in patients with NLBP (20). A 2024 systematic review and meta-analysis further synthesized 12 clinical trials of DNS/Vojta therapy for LBP and reported improvements in pain intensity, disability, and quality of life (18). However, that review included heterogeneous LBP populations—such as individuals with NLBP, postpartum LBP, lumbosciatica syndrome, scoliosis, or lumbar disc protrusion—and combined DNS with Vojta therapy as well as randomized with non-randomized controlled trials. Consequently, the specific and updated evidence for DNS-based interventions in NLBP remained uncertain. The present review therefore applied narrower eligibility criteria focused on NLBP and randomized controlled trials. In contrast to the earlier broad synthesis of pain and multiple domains of physical function, the present meta-analysis focuses on pain-related outcomes and explores whether intervention strategy, duration, weekly frequency, and session duration may contribute to variation in the effects of DNS-based interventions. This updated, diagnosis-focused synthesis was designed to clarify both the potential benefits and the current limitations of DNS for NLBP rehabilitation.

## Methods

This study was reported in accordance with the Preferred Reporting Items for Systematic Reviews and Meta-Analyses (PRISMA) guidelines, and the protocol was registered in PROSPERO (No. CRD420251152728).

### Eligibility criteria

Study selection was based on the PICOS framework, defined as follows:

Population (P): Individuals diagnosed with NLBP, defined as low back pain without an identifiable specific pathological cause (21), were eligible. No restrictions were imposed on symptom duration; therefore, studies involving acute, subacute, chronic, or mixed-duration NLBP populations were eligible. No restrictions were applied regarding age, ethnicity, sex, or occupation.
Intervention (I): Eligible interventions included DNS alone or DNS combined with a co-intervention. For combined-intervention trials, the background co-intervention was required to be provided to both groups, or both groups were required to receive equivalent background treatment while differing in the stabilization approach being evaluated.
Comparison (C): The control group received the matched background co-intervention, conventional physical therapy (e.g., hot packs, transcutaneous electrical nerve stimulation, or ultrasound), pharmacological treatment, no intervention, or another exercise program (e.g., core strength or core stability training). In add-on trials, the non-DNS co-intervention provided to the experimental group was also included in the control condition; in active-comparator trials, background care was required to be equivalent between groups.
Outcome (O): Primary outcome measures included the Visual Analogue Scale (VAS), Numerical Pain Rating Scale (NPRS), and Roland–Morris Disability Questionnaire (RMDQ). Higher VAS and NPRS scores indicated greater pain intensity, whereas lower scores or reductions from baseline indicated improvement. Among the studies meeting the inclusion criteria, only one study reported RMDQ as an outcome measure (22). Notably, this study explicitly used the RMDQ to reflect pain intensity rather than functional disability. Therefore, to ensure conceptual consistency across outcome measures, RMDQ was treated as a pain-related indicator in the present analysis. Consequently, the included studies predominantly focused on pain-related outcomes.
Study Design (S): Only randomized controlled trials were included.

### Exclusion criteria

Studies were excluded if they met any of the following conditions:

1. Single-group studies; non-randomized controlled designs; studies in which non-DNS co-interventions differed between groups in a manner that prevented estimation of the comparative effect associated with DNS; or studies in which additional unplanned interventions were introduced during the trial.
2. Studies that did not employ the predefined outcome measures, did not meet the inclusion criteria, or lacked extractable key data.
3. Studies in which DNS was not the primary intervention in the study group.
4. Case reports, review articles, animal experiments, systematic reviews, clinical guidelines, conference abstracts, duplicate publications, or studies unrelated to the research topic.

### Search strategy

Two researchers independently conducted a systematic search of PubMed, WoS, Scopus, Cochrane Library, and CNKI. The search covered all relevant studies published from the inception of each database up to February 22, 2026. To enhance the sensitivity and precision of the search, database-specific strategies combined controlled vocabulary terms, where available (e.g., MeSH terms in PubMed and the Cochrane Library), with free-text terms. For databases without a controlled vocabulary, the search syntax and relevant fields were adapted using free-text terms. Search terms included concepts related to Dynamic Neuromuscular Stabilization, such as “Muscle Strength,” “Postural Balance,” “Exercise Therapy,” and the commonly used abbreviation “DNS.” These were combined with keywords associated with low back pain, such as “Low Back Pain,” “Lumbago,” and “Low Backaches.” To minimize publication bias, gray literature databases were also searched, including OpenGrey, DART-Europe, and PROSPERO. The search was restricted to studies published in English or Chinese. Chinese-language studies were screened and extracted by reviewers proficient in Chinese. Relevant study information was translated into English and independently checked by a second reviewer to ensure accuracy.

### Study selection and data extraction

Two researchers independently screened the literature and extracted data in accordance with the predefined inclusion and exclusion criteria, with cross-checking performed to ensure accuracy. Duplicates were first removed, followed by an initial screening of titles and abstracts to exclude irrelevant or ineligible studies. Full texts of potentially relevant articles were then reviewed to determine the final set of included studies. Any disagreements were resolved through discussion; if consensus could not be reached, a third researcher was consulted for adjudication. The entire study selection process is presented in a PRISMA flow diagram to ensure transparency and systematic reporting.

Data extraction was independently conducted by two researchers using a predesigned standardized extraction form. Extracted information included the first author, publication year, sample size and mean age for each study group, intervention and comparator, intervention duration, weekly intervention frequency, duration of each session, outcome measure, and the region of the included-study authors, together with quantitative pre- and post-intervention data.

To ensure completeness and accuracy, any discrepancies during data extraction were resolved by cross-checking against the original articles. After verification, the relevant data were included in the final analysis. The entire process strictly adhered to standardized procedures to guarantee the scientific rigor and consistency of data collection and processing. When relevant outcome data or methodological information was missing or unclear, we attempted to contact the corresponding authors by email. When no response was received, the analyses were based on the information available in the published report.

### Data synthesis

All statistical analyses were performed using Review Manager 5.4 and Stata 17.0. The risk of bias of the included studies was assessed using the original Cochrane Collaboration Risk of Bias tool (RoB 1). The assessment covered seven domains: random-sequence generation, allocation concealment, blinding of participants and personnel, blinding of outcome assessment, incomplete outcome data, selective outcome reporting, and other potential sources of bias. Each domain was classified as having a low, high, or unclear risk of bias. Judgments concerning blinding, incomplete outcome data, and selective outcome reporting were made with reference to the result included in the primary meta-analysis. Two reviewers independently performed the assessments, and disagreements were resolved through discussion or consultation with a third reviewer. To account for differences in measurement instruments and effect size units across studies, standardized mean differences (SMD) were calculated for pooled analyses. Results of the meta-analysis were presented in forest plots to visually display individual study effect sizes and the overall pooled effect.

Heterogeneity among studies was assessed using Cochran's *Q* test and the I^2^ statistic. The I^2^ value describes the proportion of total variability across studies attributable to between-study heterogeneity rather than chance, with values of approximately 25%, 50%, and 75% typically representing low, moderate, and high heterogeneity, respectively. Statistical heterogeneity was considered significant when *p* < 0.10 for the Q test and/or I^2^ > 50% (23).

In addition to statistical heterogeneity, clinical and methodological heterogeneity across studies were assessed qualitatively based on study characteristics, including participant demographics, intervention protocols, and outcome measures, to ensure the appropriateness of conducting a meta-analysis.

Given the expected clinical and methodological variability among the included studies (e.g., differences in intervention protocols, participant characteristics, and outcome assessment), a random-effects model was applied when significant heterogeneity was present (*p* < 0.10 for the Q test and/or I^2^ ≥ 50%), as this model accounts for both within-study and between-study variance. Subgroup analyses were further conducted to explore potential sources of heterogeneity. When heterogeneity could not be adequately explained, a descriptive analysis was performed as a Supplementary Approach.

Potential small-study effects were explored using Begg's rank-correlation test, which evaluates the association between effect estimates and their variances. A two-sided *p*-value < 0.05 was used as the conventional threshold for statistical significance. However, because the test has limited statistical power when only a small number of studies are available, a non-significant result was considered inconclusive and was not interpreted as evidence of the absence of publication bias or small-study effects.

A leave-one-out analysis was performed by sequentially excluding each study to determine whether the pooled estimate was disproportionately influenced by any single study. This analysis was used only to assess the influence of individual studies and was not regarded as a comprehensive evaluation of the robustness or reliability of the findings. In addition, a risk-of-bias sensitivity analysis was conducted by excluding Lin et al. (2022), because the original report did not describe random allocation and this study was therefore judged to have a high risk of bias in random-sequence generation and allocation concealment.

## Results

### Search results

The initial search yielded 223 potentially relevant articles, with the Cochrane Library contributing the largest number of records. Contributions from the other databases are shown in Figure 1. After screening according to the predefined eligibility criteria, eight studies were included in the meta-analysis (22, 24–30), comprising 289 participants: 144 in the study groups and 145 in the control groups. Individual study groups included 10–30 participants. Six studies used the VAS as the outcome measure, one used the NPRS, and one used the RMDQ.

**Figure 1:**
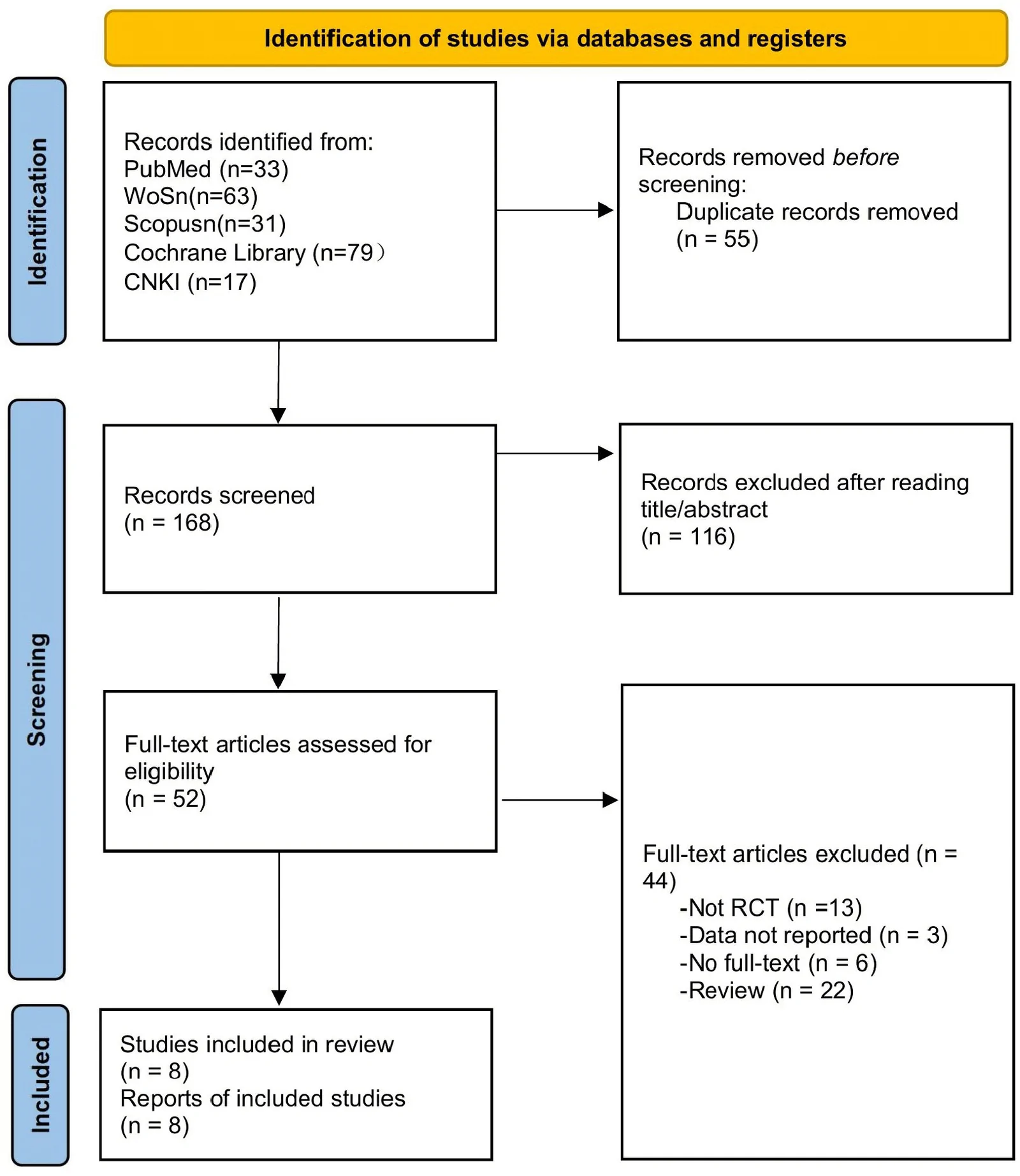
PRISMA flowchart. The PRISMA flow diagram illustrates the process of study identification and selection, from database searching, duplicate removal, and title/abstract screening, to full-text assessment and final inclusion of 8 studies.

The mean ages reported for the 16 study groups ranged from 15.94 ± 3.75 to 82.1 ± 2.2 years. Based on the group sample sizes and mean ages reported in Table 1, the estimated sample-size-weighted mean age across all 289 participants was approximately 41.6 years. The authors of four included studies were located in China, those of three studies in Iran, and those of one study in the Republic of Korea.

**Table 1:** Basic information on the included literature. Details include sample size, participant age, intervention type, intervention duration, frequency, session duration, primary outcome, and study region.

Study	N	Age	Intervention	Intervention duration (weeks)	Frequency (weekly)	Duration of each session(min)	Outcome	Region
Ghavipanje et al. 2022 (25)	20	29.25±4.14	DNS	6	6	45–60	NPRS	Iran
	20	29.35±3.48	General Exercise	6	6			
Hei et al. 2025 (26)	16	23.56±2.45	DNS and Kinesio Tape therapy	6	3	30	VAS	China
	16	24.26±1.94	Kinesio Tape therapy	6	3	The patch was individualized according to the subject’s condition.		
Huang et al. 2025 (27)	30	39.23±8.44	DNS	4	3	50	VAS	China
	30	37.43±7.38	Conventional core exercises	4	3	50		
Lin et al. 2022 (24)	22	81.4±3.2	DNS and Conventional core exercises	4	5	25	VAS	China
	22	82.1±2.2	Conventional core exercises	4	5	25		
Mousavi & Mirsafaei Rizi 2022 (28)	10	47.8±9.82	DNS	8	3	45-60	VAS	Iran
	10	42.4±11.8	Core stability	8	3	45-60		
Najafi Ghagholestani et al. 2022 (29)	12	38.19±8.54	DNS	6	3	50	VAS	Iran
	12	35.33±12.91	Non-intervention	6	3	60		
Park et al. 2021 (30)	14	15.94±3.75	DNS and Gym Ball Stability Exercise	8	3	60	VAS	Republic of Korea
	14	16.68±3.99	Gym Ball Stability Exercise	8	3	40		
Xu et al.2019 (22)	20	46.0±9.2	DNS and Physical Therapy	8	4	30	RMDQ	China
	21	48.6±8.7	Conventional core exercises and Physical Therapy	8	4	30		

DNS, dynamic neuromuscular stabilization; NPRS, numeric pain-rating scale; VAS, visual analog rating scale; RMDQ, Roland-Morris disability questionnaire.

Of the included studies, four trials applied DNS as the sole intervention in the study group, while the remaining four combined DNS with conventional interventions. Regarding intervention duration, three studies implemented a 6-week program, three studies an 8-week program, and two studies a 4-week program. In terms of frequency, five studies reported three sessions per week, while others reported four, five, and six sessions per week, respectively. The duration of each intervention session varied considerably, ranging from 25 to 60 min. The basic characteristics of the included studies are summarized in Table 1.

### Risk of bias assessment

Figure 2 summarizes the RoB 1 assessment of the included studies. Lin et al. (2022) was judged to have a high risk of bias in random-sequence generation and allocation concealment. Four studies provided insufficient information regarding allocation concealment and were therefore judged to have an unclear risk of bias in this domain. Five studies were judged to have an unclear risk of bias for blinding of participants and personnel, and six were judged to have an unclear risk for blinding of outcome assessment because the relevant procedures were insufficiently described. A study was assessed as having an unclear risk regarding incomplete results. For studies without an accessible prospective protocol or trial registration, selective outcome reporting was judged to be unclear rather than low risk.

**Figure 2:**
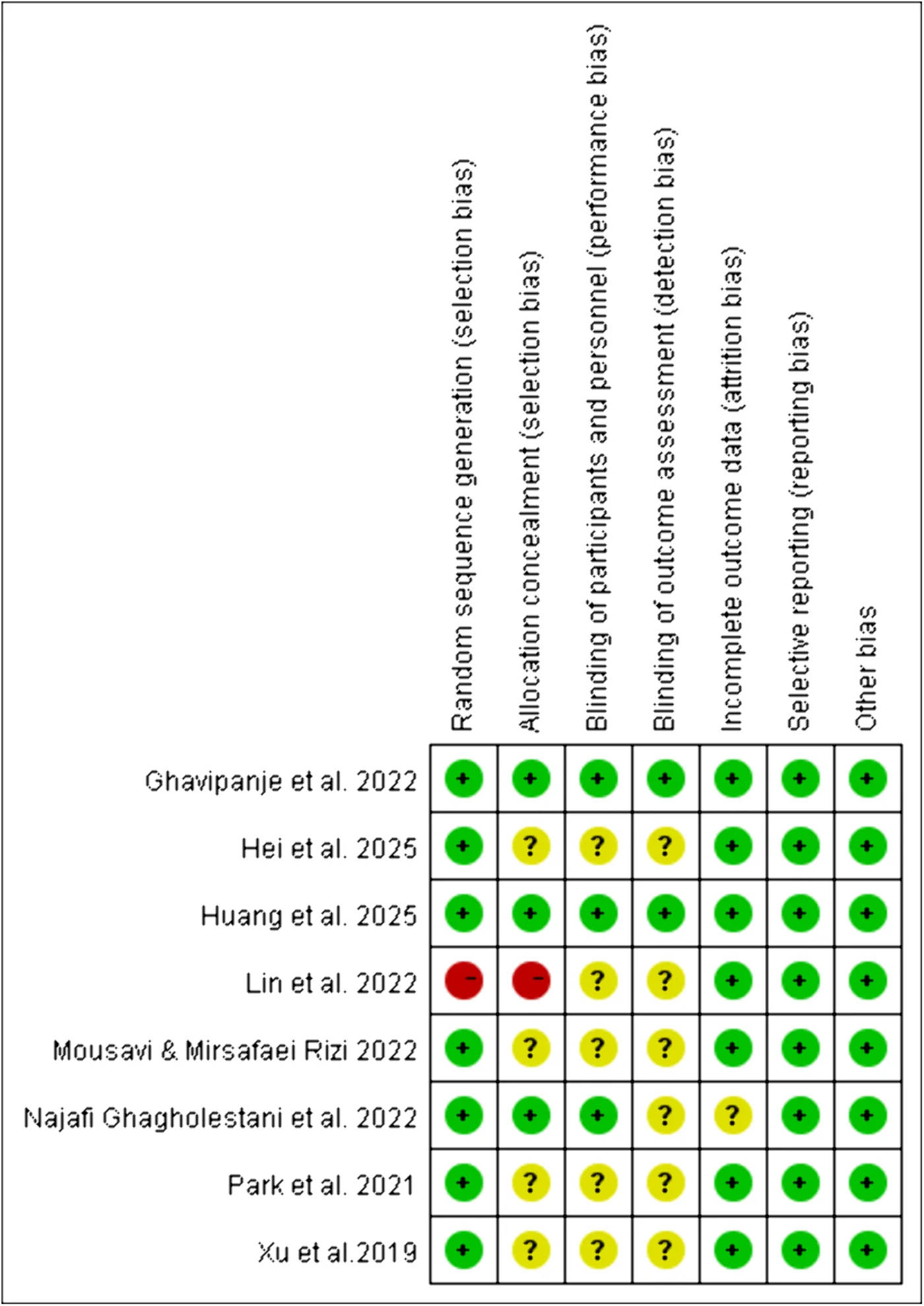
Summary of risk of bias assessment. Green indicates low risk, yellow indicates unclear risk, and red indicates high risk, across the domains of random sequence generation, allocation concealment, blinding, completeness of outcomes, and reporting bias.

Overall, the principal risk-of-bias concerns involved non-random or insufficiently described allocation procedures, inadequate reporting of allocation concealment, and limited information regarding blinding. In addition, the absence of accessible prospective protocols or trial registrations for some studies prevented selective outcome reporting from being confidently excluded. These methodological limitations may have introduced selection, performance, detection, or reporting bias and may partly explain the heterogeneity observed in the pooled analysis.

Overall, the principal risk-of-bias concerns involved non-random or insufficiently described allocation procedures, inadequate reporting of allocation concealment, and limited information regarding blinding. In addition, the absence of accessible prospective protocols or trial registrations for some studies prevented selective outcome reporting from being confidently excluded. These methodological limitations may have introduced selection, performance, detection, or reporting bias and may partly explain the heterogeneity observed in the pooled analysis.

### Meta-analysis

By comparing the post-intervention changes in mean values and standard deviations between the study and control groups, this study evaluated the therapeutic effect of DNS-based interventions on NLBP. All statistical inferences were conducted using a random-effects model. As shown in Figure 3, the pooled effect size indicated that DNS intervention significantly alleviated pain symptoms. The combined SMD was −1.17 (95% CI: −1.85 to −0.48), suggesting that the study group reported significantly lower pain levels than the control group. However, substantial heterogeneity was detected across the eight included studies (I^2^ = 85%, *p* < 0.01), indicating variability in results between studies.

**Figure 3:**
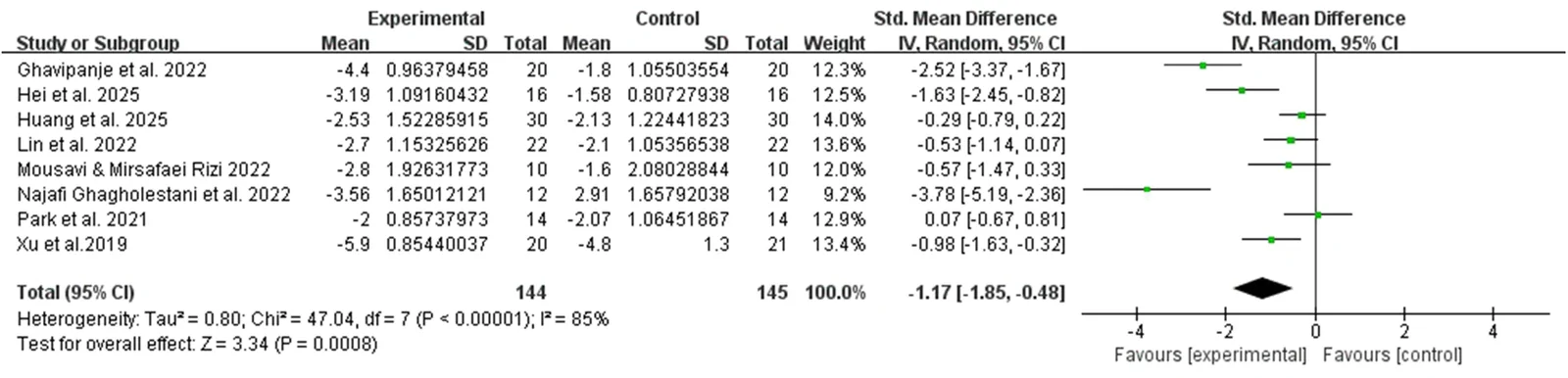
Forest plot of the effects of DNS-based interventions on pain-related outcomes in individuals with NLBP. Effect estimates are presented as SMD with 95% CI and were pooled using a random-effects model. Squares represent individual-study estimates, with their sizes reflecting study weights; horizontal lines indicate 95% CI, and the diamond represents the pooled estimate. Negative SMD values indicate lower pain-related scores and favor the DNS-based intervention, whereas positive values favor the comparator.

### Subgroup analysis

Given the substantial heterogeneity among the included studies, subgroup analyses were conducted to explore potential sources of variability (Figures 4–7). Specifically, studies were first categorized according to the intervention strategies in the study group: DNS-only interventions (study group receiving DNS training alone) and combined interventions (study group receiving DNS training in addition to other treatments). Second, further analyses were performed based on intervention duration (4 weeks, 6 weeks, or 8 weeks), intervention frequency (< 4 sessions/week vs. ≥ 4 sessions/week), and session duration (< 45 min vs. ≥ 45 min per session).

**Figure 4:**
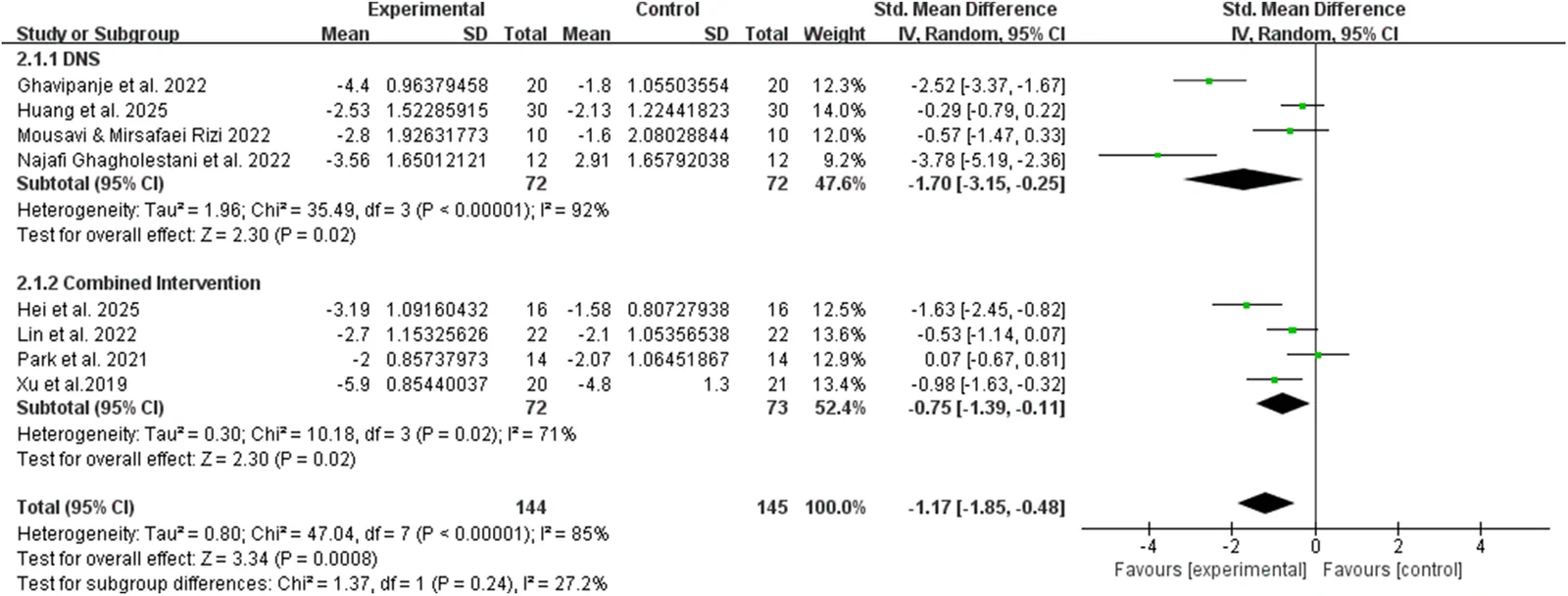
Subgroup analysis according to intervention strategy. Studies were classified as DNS-only interventions or combined interventions in which DNS was administered with an additional treatment. Effect estimates are presented as SMD with 95% CI and were pooled using a random-effects model. Negative SMD values indicate lower pain-related scores and favor the DNS-based intervention, whereas positive values favor the comparator. The subgroup analysis should be interpreted as exploratory.

**Figure 5:**
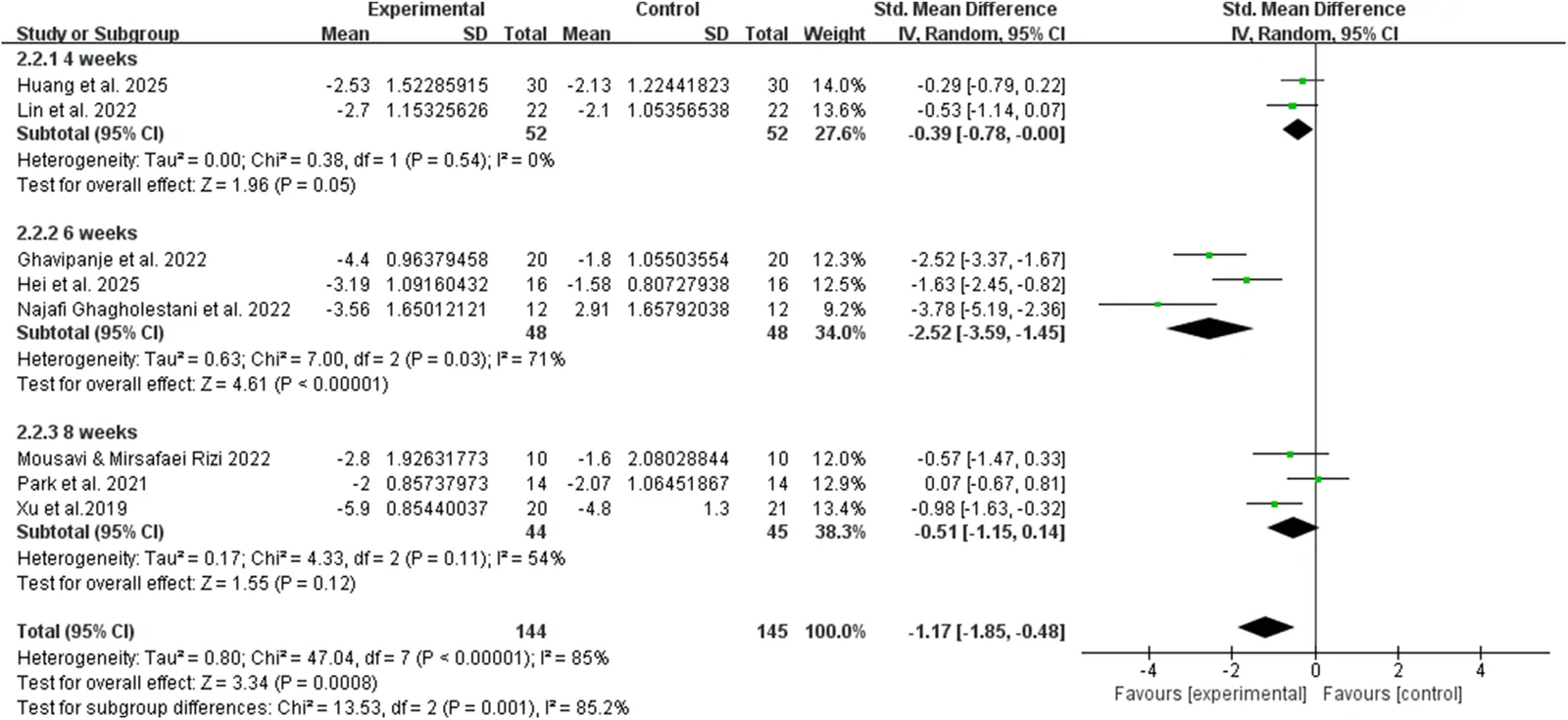
Subgroup analysis according to intervention duration. Studies were classified according to intervention durations of 4, 6, or 8 weeks. Study-specific and subgroup-pooled effects are presented as SMD with 95% CI using a random-effects model. Negative SMD values indicate lower pain-related scores and favor the DNS-based intervention, whereas positive values favor the comparator. The subgroup analysis should be interpreted as exploratory.

**Figure 6:**
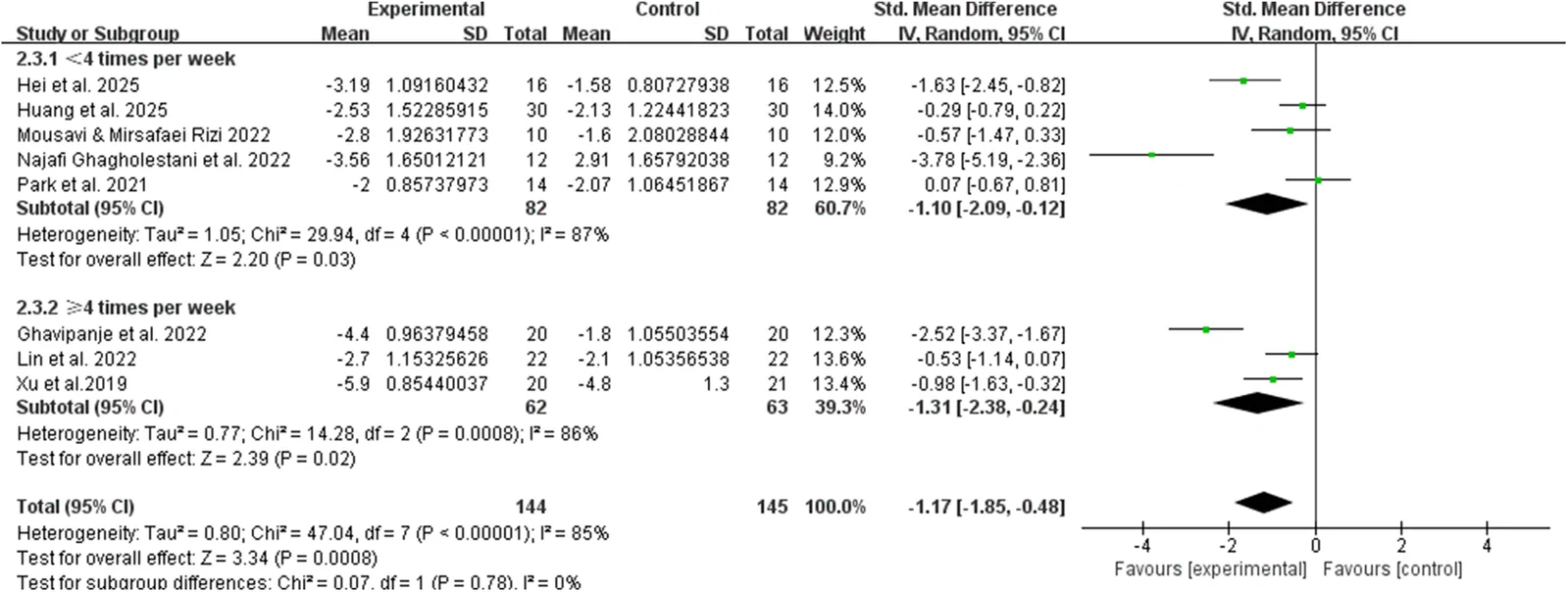
Subgroup analysis according to weekly intervention frequency. Studies were classified as providing fewer than four sessions per week or at least four sessions per week. Study-specific and subgroup-pooled effects are presented as SMD with 95% CI using a random-effects model. Negative SMD values indicate lower pain-related scores and favor the DNS-based intervention, whereas positive values favor the comparator. The subgroup analysis should be interpreted as exploratory.

**Figure 7:**
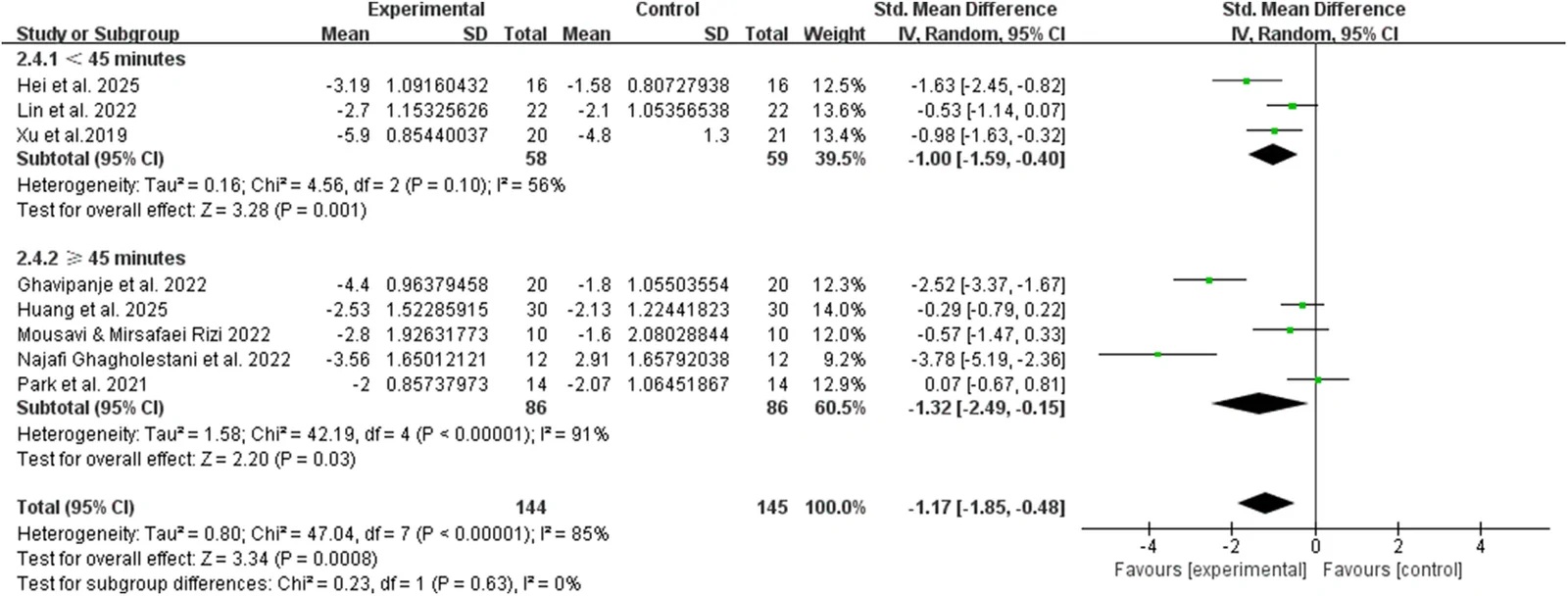
Subgroup analysis according to session duration. Studies were classified as providing sessions lasting less than 45 min or at least 45 min. Study-specific and subgroup-pooled effects are presented as SMD with 95% CI using a random-effects model. Negative SMD values indicate lower pain-related scores and favor the DNS-based intervention, whereas positive values favor the comparator. The subgroup analysis should be interpreted as exploratory.

Subgroup analyses according to intervention strategy, intervention duration, weekly frequency, and session duration did not clearly explain the observed heterogeneity. Substantial heterogeneity persisted in most subgroups. Although heterogeneity was reduced to I^2^ = 0% in the 4-week subgroup, this subgroup contained only two studies, making the estimate imprecise and potentially unstable. Given the small number of studies within each subgroup, these analyses should be considered exploratory. Therefore, the present findings do not demonstrate that these intervention characteristics are unrelated to the observed variability; rather, the available evidence was insufficient to identify a definitive source of heterogeneity.

### Assessment of small-study effects and sensitivity analysis

Begg's rank-correlation test yielded a *p*-value of 0.063. However, because only eight studies were included, the test had limited statistical power to detect small-study effects. The assessment was therefore considered inconclusive, and publication bias or small-study effects could neither be confirmed nor excluded.

Furthermore, a leave-one-out sensitivity analysis was performed to examine the influence of individual studies on the pooled estimate. When Lin et al. (2022), which was judged to have a high risk of bias because of its non-random allocation procedure, was excluded, the pooled effect remained statistically significant and its direction was unchanged. The direction and statistical significance of the pooled effect also remained broadly consistent when the other studies were excluded individually. Nevertheless, this stability does not resolve the substantial between-study heterogeneity. The sensitivity analysis therefore supports the stability of the average effect estimate but does not establish that the magnitude of benefit is consistent across populations or intervention protocols (Figure 8).

**Figure 8:**
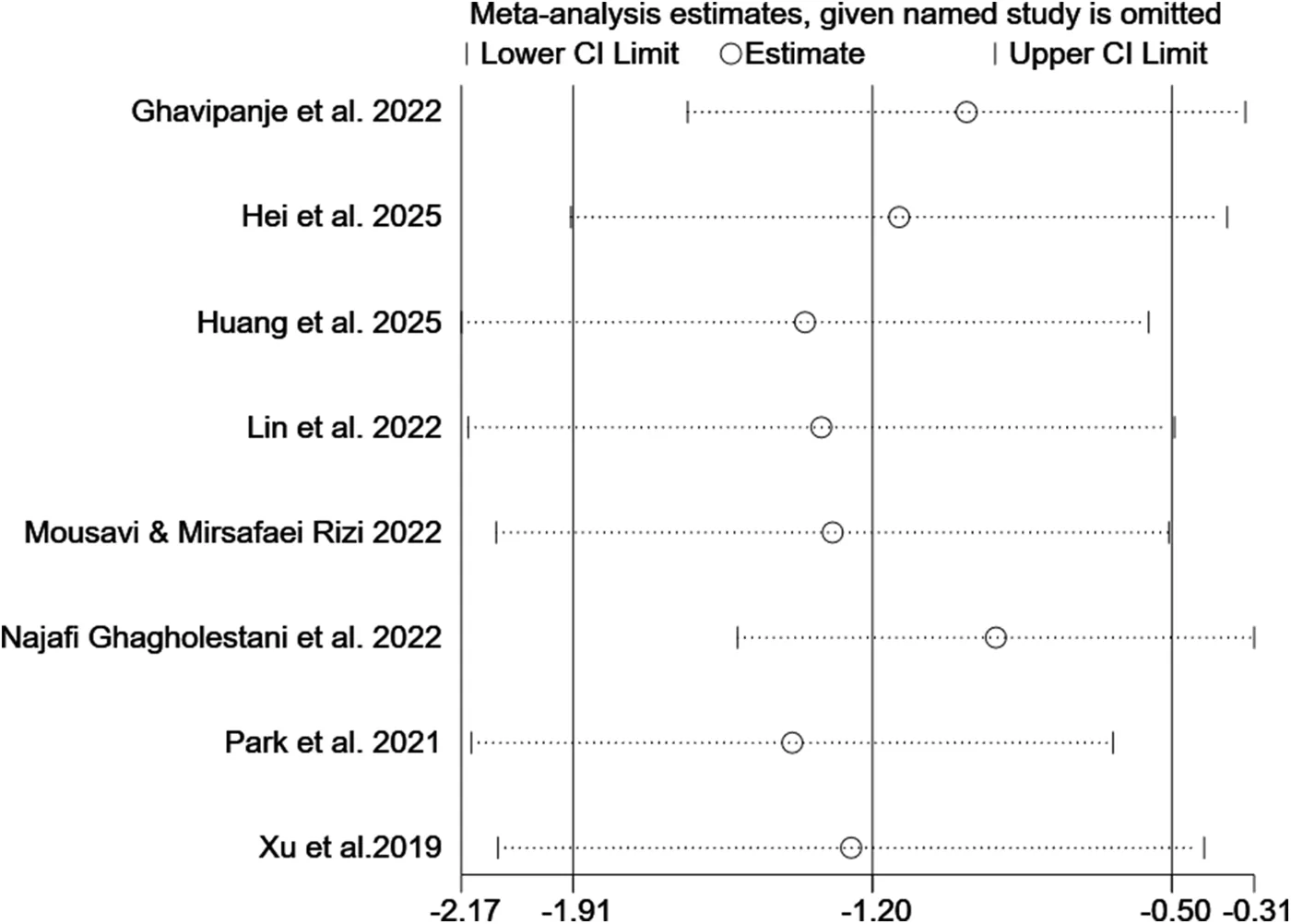
Leave-one-out analysis. Each study was sequentially excluded to examine its individual influence on the pooled effect estimate and 95% confidence interval. The analysis indicates whether the result was dominated by a single study but does not evaluate other sources of clinical, methodological, or model-related uncertainty.

## Discussion

This systematic review and meta-analysis evaluated the effects of DNS-based interventions in individuals with nonspecific low back pain. The pooled analysis suggested a reduction in pain [SMD = −1.17, 95% CI (−1.85, −0.48)]; however, the substantial heterogeneity indicates that this estimate represents an average across diverse study conditions rather than a uniform treatment effect. In addition, the overall methodological quality of the included studies was moderate, with unclear or high risk of bias in randomization, allocation concealment, and blinding in several studies. Consequently, the magnitude and clinical applicability of the pooled effect should be interpreted cautiously.

Low back pain is one of the leading causes of disability worldwide and ranks sixth in the overall global burden of disease, yet a substantial proportion of patients do not receive systematic professional treatment (31). The occurrence of NLBP is associated with multiple risk factors, including unfavorable lifestyle habits such as prolonged sitting, frequent heavy lifting, and carrying, as well as psychosocial factors such as depression and smoking (32). Other studies have also indicated that demographic characteristics such as sex and obesity may contribute to its development (8). Although its pathological mechanisms are multifactorial, disordered neuromuscular control of lumbar muscles, muscle fatigue, and reduced contractile strength are considered to be closely related. Evidence suggests that, under spinal pain conditions, the central nervous system may undergo adaptive changes in its control of trunk muscles (33). Moreover, compared with healthy individuals, patients with NLBP frequently exhibit impaired proprioceptive function, which is essential for maintaining normal motor control. When proprioceptive input becomes inconsistent with motor intention, cortical mechanisms responsible for monitoring sensory mismatches may be activated, thereby inducing pathological pain (34). At the same time, patients with NLBP often present with respiratory dysfunction, characterized by reduced diaphragmatic mobility, insufficient respiratory muscle endurance (12), and atrophy of the transversus abdominis (35), all of which may compromise trunk and spinal stability and functional performance.

The significant pain reduction observed in this meta-analysis may be explained by several underlying mechanisms of DNS. The core objective of DNS is to optimize the distribution of muscular forces across spinal segments and joints while simultaneously correcting and improving breathing strategies (14). This training approach effectively integrates innate normative motor patterns into spontaneous activity, thereby restoring the coordination of kinematic responses and promoting synergistic activation of the abdominal musculature and the spinal autochthonous muscles (16). Systematic DNS-based training plays a critical role in mitigating related risk factors, correcting maladaptive postural habits in daily life, enhancing the regulation of intra-abdominal pressure stability, and reinforcing the dynamic interactions between the thoracic and pelvic regions (17, 36). Moreover, by facilitating the execution of optimal muscular movement strategies, DNS helps meet the mechanical demands of joints during functional activities, prevents excessive joint loading caused by overactivation of myofascial structures, and protects against overstretching or tightening of periarticular soft tissues (37, 38). The pooled SMD indicated a statistically favorable effect of DNS-based interventions. However, because different outcome instruments and potentially different constructs were combined, the magnitude of the SMD cannot be directly translated into a clinically meaningful change on a familiar pain scale. Therefore, the clinical importance of the observed effect remains uncertain.

The diaphragm, as a key deep muscle for maintaining postural stability, fulfills dual functions in both respiration and postural control (39). When breathing patterns become abnormal, its function may be compromised, thereby exerting a negative influence on the overall quality of motor patterns (18). Consequently, in alleviating pain among patients with NLBP, it is of particular importance to promote the establishment of normal breathing patterns and to progressively restore the role of the diaphragm in postural control (39). Similar to yoga training, DNS provides a systematic respiratory intervention program for patients with low back pain by integrating postural adjustments with core stability training (40). In addition, DNS can induce reflexive synergistic activation of the diaphragm and other core stabilizing muscles through subconscious stimulation of specific regions (41), thereby generating the stable torque required to support joint movement. However, variations in how these mechanisms are implemented across studies may partly explain the substantial heterogeneity observed in the present analysis.

The substantial heterogeneity observed in the overall analysis (I^2^ = 85%) indicates that the pooled SMD should be interpreted as an average effect across clinically and methodologically diverse studies rather than as a uniform treatment effect. Several factors may have contributed to this variability. First, the DNS interventions differed substantially: four studies evaluated DNS alone, whereas four combined DNS with another therapeutic intervention. Intervention duration ranged from 4 to 8 weeks, weekly frequency from 3 to 6 sessions, and session duration from 25 to 60 min. Comparator conditions also varied and included general exercise, core stability training, Kinesio taping, conventional physical therapy, gym-ball stability exercise, and no intervention. Second, the mean ages of the study groups ranged from 15.94 to 82.1 years, indicating considerable variation in participant age. Third, different outcome instruments were used, including the VAS, NPRS, and RMDQ. Standardization of effect sizes may reduce scale-related differences but does not eliminate differences in the constructs measured. Finally, variation in randomization, allocation concealment, blinding, baseline symptom severity, treatment adherence, and implementation of the DNS protocols may also have influenced the observed effects.

In addition, comorbidities such as obesity, metabolic disorders, or psychological conditions may influence treatment outcomes by affecting neuromuscular control or adherence to exercise (42, 43). Furthermore, individual characteristics such as age, sex, occupational factors, and prior exercise habits (8, 17, 44) may also contribute to variability in treatment response. As most included studies did not adequately control for these factors, their potential confounding effects may partly explain the substantial heterogeneity observed in this meta-analysis.

This study has several limitations related to the systematic review process and study conduct. First, although a comprehensive search strategy was applied, only a limited number of studies met the inclusion criteria, which may restrict the statistical power of the meta-analysis and the reliability of subgroup analyses. Second, substantial heterogeneity was observed across studies, and although a random-effects model and subgroup analyses were employed, the sources of heterogeneity could not be fully explained, which may affect the stability and interpretability of the pooled estimates. Third, subgroup analyses were based on a small number of studies within each category and should therefore be considered exploratory. The leave-one-out analysis assessed only the influence of individual studies and should not be interpreted as establishing the overall robustness of the pooled result. Additional targeted sensitivity analyses excluding the study that used the RMDQ, studies using combined interventions, or studies with less comparable control conditions were not conducted. Therefore, the extent to which outcome selection, intervention composition, comparator heterogeneity, and model uncertainty influenced the pooled estimate remains uncertain.

In addition, the assessment of publication bias and small-study effects was inconclusive because only eight studies were available, resulting in limited statistical power for Begg's test. Finally, although efforts were made to ensure methodological rigor (e.g., predefined protocol and standardized data extraction), the review process may still be subject to inherent limitations, such as selection bias and incomplete reporting in the original studies. Future research should aim to include a larger body of high-quality evidence to enable more robust and reliable meta-analytic conclusions.

## Conclusions

This meta-analysis suggests that DNS-based interventions may reduce pain-related outcomes in individuals with NLBP. However, this finding should not be interpreted as definitive evidence of the magnitude or clinical importance of the effect. The synthesis was based on only eight relatively small studies, resulting in limited statistical precision. Substantial between-study heterogeneity was present, and several studies had unclear or high risk of bias in randomization, allocation concealment, and blinding. Differences in participant characteristics, DNS protocols, combined interventions, comparator conditions, and outcome instruments—including the combination of pain-intensity measures with the RMDQ—further limit the comparability and direct clinical interpretation of the pooled estimate. In addition, the assessment of publication bias and small-study effects was inconclusive because of the limited number of studies.

Taken together, these limitations preclude firm conclusions regarding the magnitude, clinical importance, and generalizability of the observed effect. The findings should therefore be regarded as preliminary. Future adequately powered, prospectively registered randomized controlled trials using standardized DNS protocols, clearly defined comparator conditions, consistent pain-specific outcome measures, and rigorous methods are needed to determine whether DNS provides clinically meaningful benefits for individuals with NLBP.

## Data Availability

The original contributions presented in the study are included in the article/Supplementary Material, further inquiries can be directed to the corresponding author.
