# Supplementary material for: Effectiveness of dynamic neuromuscular stabilization-based interventions for nonspecific low back pain: a systematic review and meta-analysis
Source: Front Med (Lausanne). 2026 Sep 28;13:1926914. doi: 10.3389/fmed.2026.1926914 (PMC13655038; doi:10.3389/fmed.2026.1926914)
Supplement: Supplementary file 1 [file Supplementaryfile1.docx]

**Search strategy**

**Pubmed**

((((((("Muscle Strength"[Mesh]) AND ("Postural Balance"[Mesh])) AND ("Exercise Therapy"[Mesh])) AND ("Low Back Pain"[Mesh])) OR (((((("Muscle Strength"[Mesh]) OR (Strength, Muscle[Title/Abstract])) OR (Arthrogenic Muscle Inhibition[Title/Abstract])) OR (Arthrogenic Muscle Inhibitions[Title/Abstract])) OR (Inhibition, Arthrogenic Muscle[Title/Abstract])) OR (Muscle Inhibition, Arthrogenic[Title/Abstract]))) AND (((((((((((((((((("Postural Balance"[Mesh]) OR (Posture Balance)) OR (Balance, Posture)) OR (Posture Balances)) OR (Posture Equilibrium)) OR (Equilibrium, Posture)) OR (Posture Equilibriums)) OR (Musculoskeletal Equilibrium)) OR (Equilibrium, Musculoskeletal)) OR (Postural Equilibrium)) OR (Equilibrium, Postural)) OR (Balance, Postural)) OR (Postural Control)) OR (Control, Postural)) OR (Postural Controls)) OR (Posture Control)) OR (Control, Posture)) OR (Posture Controls))) AND (((((((((((("Exercise Therapy"[Mesh]) OR (Rehabilitation Exercise)) OR (Exercise, Rehabilitation)) OR (Exercises, Rehabilitation)) OR (Rehabilitation Exercises)) OR (Therapy, Exercise)) OR (Exercise Therapies)) OR (Therapies, Exercise)) OR (Remedial Exercise)) OR (Exercise, Remedial)) OR (Exercises, Remedial)) OR (Remedial Exercises))) AND (((((((((((((((((((((((((((((("Low Back Pain"[Mesh]) OR (Back Pain, Low)) OR (Back Pains, Low)) OR (Low Back Pains)) OR (Pain, Low Back)) OR (Pains, Low Back)) OR (Low Back Ache)) OR (Ache, Low Back)) OR (Aches, Low Back)) OR (Back Ache, Low)) OR (Back Aches, Low)) OR (Low Back Aches)) OR (Low Backache)) OR (Backache, Low)) OR (Backaches, Low)) OR (Low Backaches)) OR (Lower Back Pain)) OR (Back Pain, Lower)) OR (Back Pains, Lower)) OR (Lower Back Pains)) OR (Pain, Lower Back)) OR (Pains, Lower Back)) OR (Lumbago)) OR (Low Back Pain, Mechanical)) OR (Mechanical Low Back Pain)) OR (Low Back Pain, Posterior Compartment)) OR (Low Back Pain, Postural)) OR (Postural Low Back Pain)) OR (Low Back Pain, Recurrent)) OR (Recurrent Low Back Pain))

**WoS**

TS=(Muscle Strength OR Arthrogenic Muscle Inhibition OR Arthrogenic Muscle Inhibitions)

TS=(Postural Balance OR Posture Balance OR Posture Balances OR Posture Equilibrium OR Posture Equilibriums OR Musculoskeletal Equilibrium OR Postural Equilibrium OR Postural Control OR Postural Controls OR Posture Control OR Posture Controls)

TS=(Exercise Therapy OR Rehabilitation Exercise OR Rehabilitation Exercises OR Exercise Therapies OR Remedial Exercise OR Remedial Exercises)

TS=(Low Back Pain OR Low Back Pains OR Low Back Ache OR Low Back Aches OR Low Backache OR Low Backaches OR Lower Back Pain OR Lower Back Pains OR Lumbago OR Mechanical Low Back Pain OR Postural Low Back Pain OR Recurrent Low Back Pain)

**Scopus**

Muscle Strength OR "Strength, Muscle" OR "Arthrogenic Muscle Inhibition" OR "Arthrogenic Muscle Inhibitions" OR "Inhibition, Arthrogenic Muscle" OR "Muscle Inhibition, Arthrogenic"

Postural Balance OR "Posture Balance" OR "Balance, Posture" OR "Posture Balances" OR "Posture Equilibrium" OR "Equilibrium, Posture" OR "Posture Equilibriums" OR "Musculoskeletal Equilibrium" OR "Equilibrium, Musculoskeletal" OR "Postural Equilibrium" OR "Equilibrium, Postural" OR "Balance, Postural" OR "Postural Control" OR "Control, Postural" OR "Postural Controls" OR "Posture Control" OR "Control, Posture" OR "Posture Controls"

Exercise Therapy OR "Rehabilitation Exercise" OR "Remedial Exercise" OR "Therapy, Exercise"

Low Back Pain OR "Back Pain, Low" OR "Back Pains, Low" OR "Low Back Pains" OR "Pain, Low Back" OR "Pains, Low Back" OR "Low Back Ache" OR "Ache, Low Back" OR "Aches, Low Back" OR "Back Ache, Low" OR "Back Aches, Low" OR "Low Back Aches" OR "Low Backache" OR "Backache, Low" OR "Backaches, Low" OR "Low Backaches" OR "Lower Back Pain" OR "Back Pain, Lower" OR "Back Pains, Lower" OR "Lower Back Pains" OR "Pain, Lower Back" OR "Pains, Lower Back" OR "Lumbago" OR "Low Back Pain, Mechanical" OR "Mechanical Low Back Pain" OR "Low Back Pain, Posterior Compartment" OR "Low Back Pain, Postural" OR "Postural Low Back Pain" OR "Low Back Pain, Recurrent" OR "Recurrent Low Back Pain"

**Cochrane Library**

(Muscle Strength):ti,ab,kw OR (Strength, Muscle):ti,ab,kw OR (Arthrogenic Muscle Inhibition):ti,ab,kw OR (Arthrogenic Muscle Inhibitions):ti,ab,kw OR (Inhibition, Arthrogenic Muscle):ti,ab,kw OR (Muscle Inhibition, Arthrogenic):ti,ab,kw

(Postural Balance):ti,ab,kw OR (Posture Balance):ti,ab,kw OR (Balance, Posture):ti,ab,kw OR (Posture Balances):ti,ab,kw OR (Posture Equilibrium):ti,ab,kw OR (Equilibrium, Posture):ti,ab,kw OR (Posture Equilibriums):ti,ab,kw OR (Musculoskeletal Equilibrium):ti,ab,kw OR (Equilibrium, Musculoskeletal):ti,ab,kw OR (Postural Equilibrium):ti,ab,kw OR (Equilibrium, Postural):ti,ab,kw OR (Balance, Postural):ti,ab,kw OR (Postural Control):ti,ab,kw OR (Control, Postural):ti,ab,kw OR (Postural Controls):ti,ab,kw OR (Posture Control):ti,ab,kw OR (Control, Posture):ti,ab,kw OR (Posture Controls):ti,ab,kw

( Therapy, Exercise):ti,ab,kw OR ( Exercises, Remedial):ti,ab,kw OR ( Therapies, Exercise):ti,ab,kw OR ( Exercise, Rehabilitation):ti,ab,kw OR ( Exercise, Remedial):ti,ab,kw OR ( Exercises, Rehabilitation):ti,ab,kw OR ( Rehabilitation Exercises):ti,ab,kw OR ( Rehabilitation Exercise):ti,ab,kw OR ( Remedial Exercise):ti,ab,kw OR ( Exercise Therapies):ti,ab,kw OR ( Remedial Exercises):ti,ab,kw

( Low Back Pain):ti,ab,kw OR (Back Pain, Low):ti,ab,kw OR (Back Pains, Low):ti,ab,kw OR (Low Back Pains):ti,ab,kw OR (Pain, Low Back):ti,ab,kw OR (Pains, Low Back):ti,ab,kw OR (Low Back Ache):ti,ab,kw OR (Ache, Low Back):ti,ab,kw OR (Aches, Low Back):ti,ab,kw OR (Back Ache, Low):ti,ab,kw OR (Back Aches, Low):ti,ab,kw OR (Low Back Aches):ti,ab,kw OR (Low Backache):ti,ab,kw OR (Backache, Low):ti,ab,kw OR (Backaches, Low):ti,ab,kw OR (Low Backaches):ti,ab,kw OR (Lower Back Pain):ti,ab,kw OR (Back Pain, Lower):ti,ab,kw OR (Back Pains, Lower):ti,ab,kw OR (Lower Back Pains):ti,ab,kw OR (Pain, Lower Back):ti,ab,kw OR (Pains, Lower Back):ti,ab,kw OR (Lumbago):ti,ab,kw OR (Low Back Pain, Mechanical):ti,ab,kw OR (Mechanical Low Back Pain):ti,ab,kw OR (Low Back Pain, Posterior Compartment):ti,ab,kw OR (Low Back Pain, Postural):ti,ab,kw OR (Postural Low Back Pain):ti,ab,kw OR (Low Back Pain, Recurrent):ti,ab,kw OR (Recurrent Low Back Pain):ti,ab,kw

**CNKI**

动态神经稳定 + DNS

腰痛 + 下背痛 + 慢性腰痛
