# Supplementary material for: Effectiveness of dynamic neuromuscular stabilization-based interventions for nonspecific low back pain: a systematic review and meta-analysis
Source: Front Med (Lausanne). 2026 Sep 28;13:1926914. doi: 10.3389/fmed.2026.1926914 (PMC13655038; doi:10.3389/fmed.2026.1926914)
Supplement: Supplementary file 2 [file Table1.docx]

**The data of Meta analysis**

|  | Experimental group | | | Control group | | |
| --- | --- | --- | --- | --- | --- | --- |
| study | m1 | sd1 | n1 | m2 | sd2 | n2 |
| Ghavipanje et al. 2022 | -4.4 | 0.963794584 | 20 | -1.8 | 1.055035544 | 20 |
| Hei et al. 2025 | -3.19 | 1.091604324 | 16 | -1.58 | 0.807279382 | 16 |
| Huang et al. 2025 | -2.53 | 1.522859153 | 30 | -2.13 | 1.224418229 | 30 |
| Lin et al. 2022 | -2.7 | 1.153256259 | 22 | -2.1 | 1.053565375 | 22 |
| Mousavi & Mirsafaei Rizi 2022 | -2.8 | 1.926317731 | 10 | -1.6 | 2.080288442 | 10 |
| Najafi Ghagholestani et al. 2022 | -3.56 | 1.650121208 | 12 | 2.91 | 1.657920384 | 12 |
| Park et al. 2021 | -2 | 0.857379729 | 14 | -2.07 | 1.064518671 | 14 |
| Xu et al.2019 | -5.9 | 0.854400375 | 20 | -4.8 | 1.3 | 21 |
